# Surface Structuring with Polarization-Singular Femtosecond Laser Beams Generated by a q-plate

**DOI:** 10.1038/srep42142

**Published:** 2017-02-07

**Authors:** Jijil JJ Nivas, Filippo Cardano, Zhenming Song, Andrea Rubano, Rosalba Fittipaldi, Antonio Vecchione, Domenico Paparo, Lorenzo Marrucci, Riccardo Bruzzese, Salvatore Amoruso

**Affiliations:** 1 Dipartimento di Fisica “Ettore Pancini”, Università di Napoli Federico II, Complesso Universitario di Monte S. Angelo, Via Cintia, I-80126 Napoli, Italy; 2 CNR-SPIN UOS Napoli, Complesso Universitario di Monte S. Angelo, Via Cintia, I-80126 Napoli, Italy; 3 Department of Physics, School of Science, Tianjin Polytechnic University, Binshuixi Road 399#, Xiqing District, Tianjin, 300387, P. R. China; 4 CNR-SPIN, UOS Salerno, Via Giovanni Paolo II 132, I-84084 Fisciano, Italy; 5 National Research Council, Institute of Applied Science & Intelligent Systems (ISASI) ‘E. Caianiello’, Via Campi Flegrei 34, 80078 Pozzuoli (NA), Italy

## Abstract

In the last few years femtosecond optical vortex beams with different spatial distributions of the state of polarization (e.g. azimuthal, radial, spiral, etc.) have been used to generate complex, regular surface patterns on different materials. Here we present an experimental investigation on direct femtosecond laser surface structuring based on a larger class of vector beams generated by means of a q-plate with topological charge *q* = +1/2. In fact, voltage tuning of q-plate optical retardation allows generating a family of ultrashort laser beams with a continuous spatial evolution of polarization and fluence distribution in the focal plane. These beams can be thought of as a controlled coherent superposition of a Gaussian beam with uniform polarization and a vortex beam with a radial or azimuthal state of polarization. The use of this family of ultrashort laser beams in surface structuring leads to a further extension of the achievable surface patterns. The comparison of theoretical predictions of the vector beam characteristics at the focal plane and the generated surface patterns is used to rationalize the dependence of the surface structures on the local state of the laser beam, thus offering an effective way to either design unconventional surface structures or diagnose complex ultrashort laser beams.

Surface morphology plays a crucial role in defining many properties (e.g., optical, mechanical, chemical, biological, wetting, etc.) of a material. Over the last decades, laser processing has attracted continuously increasing attention due to the capability to process any kind of material with excellent quality^1^,^2^. In recent years, ultrashort laser beams are constantly showing impressive scientific achievements in the fabrication of a plurality of surface structures, including e.g. laser-induced periodic surface structures (LIPSS), cones arrays, random patterns, and so forth^1^,^2^,^3^,^4^. A fascinating aspect of fs laser surface structuring of metals and semiconductors is the formation of quasi-periodic surface structures preferentially oriented along the direction normal to laser polarization and characterized by a period lower than laser wavelength, typically indicated as ripples^3^,^4^. In semiconductors (e.g., Si and InP), the laser surface structuring is accompanied by the progressive formation of other quasi-periodic structures, dubbed as grooves, preferentially oriented along laser polarization and with an above-wavelength period^5^,^6^,^7^,^8^,^9^. These structures attract particular interest because of their large promise to generate manifold functional surfaces for various applications^1^,^2^,^3^,^4^,^10^. The creation of surface structures depends on several experimental parameters including, e.g., wavelength *λ*, fluence *F*, number *N* of femtosecond (fs) laser pulses hitting the target surface, and material properties, but the key-factor is related to the state of polarization (SoP) of the laser beam. This strong influence is particularly evidenced by the preferential alignment as well as the degree of order/disorder of the emerging surface patterns. Generally, the surface structuring phenomena are investigated by using laser beams with a transverse Gaussian intensity spatial profile and a spatially homogeneous distribution of the polarization (linear, elliptical and circular, e.g.).

The use of optical cylindrical vector beams with fs pulse duration has been demonstrated as a striking laser surface fabrication method for the generation of surface patterns with axial symmetry (e.g., azimuthal, radial, spiral, etc.)^11^,^12^,^13^,^14^,^15^,^16^, thanks to their numerous spatially variant SoP^17^. Moreover, the direct relationship of surface structures orientation and morphological features with laser light polarization and fluence has also been demonstrated as an effective, direct way for the characterization of intense optical vector beams^18^,^19^,^20^,^21^. Going beyond standard radially and azimuthally polarized vortex beams, in this paper we report on direct fs laser surface structuring of silicon using a larger class of vector beams generated by means of a *q-plate* with topological charge *q* = +1/2, obtained by varying the optical birefringent retardation *δ* of such device^22^. A q-plate is made essentially of a thin layer of liquid crystals, whose optic axes are arranged so as to form a singular pattern with topological charge *q*^23^. Such layer has a homogeneous thickness, hence the birefringent retardation is uniform point by point along the plate. Importantly, by applying a tunable electric field to the outer faces of the cell it is possible to vary *δ*^24^, thus controlling the spin-orbit interaction mediated by the device. Although accessing the full range of allowed optical retardations has proved to be useful for different applications, as for instance the quantum simulation of topological phases^25^, the opportunity of finely controlling *δ* has not been fully explored hitherto. Here we demonstrate a novel application of this concept in the context of direct fs laser surface structuring. A tuned q-plate (*δ* = *π*) shined with linearly polarized Gaussian beams allows generating cylindrically symmetric vortex beams, e.g. radial and azimuthal. Detuning the q-plate, that is varying *δ* away from to its optimal value, a variety of fs laser beams with an asymmetric spatial distribution of intensity and SoP in the focal plane are generated, which in turn allow achieving ‘lopsided’ surface patterns on a silicon target. Silicon is selected as target because it is a case study semiconductor material with a wide range of applications (e.g., in mechanical, optical and electronic devices) and since it shows several kinds of surface structures^3^,^26^. The various features of the surface patterns and the vector beams characteristics at the focal plane are compared, thus demonstrating how the spatial variation of the local state of the laser beam offers an effective way to both design unconventional, asymmetric surface structures and characterize complex ultrashort laser beams.

## Results

### Principle

**Figure 1:**
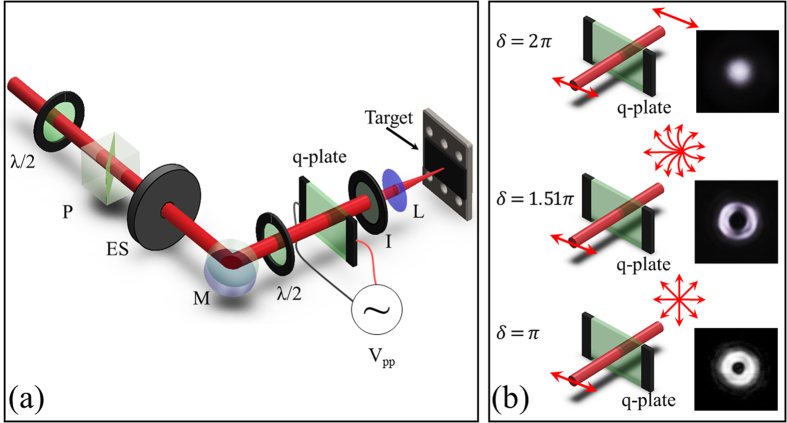
(**a**) Schematic of the experimental setup used for direct laser surface structuring with fs laser beams generated by a *q-plate*; λ/2 = half-wave plate, P = polarizer, ES = electro-mechanical shutter; M = mirror; I = iris; L = lens (**b**) Three examples of the q-plate configuration for various optical retardations *δ. δ* = 2π leads to a linearly polarized Gaussian beam at the output of the q-plate (upper panel); *δ* = π corresponds to the q-plate tuning condition leading to the generation of optical vortex beams (lower panel). *δ* = 1.51 π shows an example of q-plate tuning condition leading to the generation of a generalized vector beam with an asymmetric spatial distribution of the SoP (central panel). The arrows schematically indicate the SoP input and output beams at the q-plate. The right images show examples of the spatial profiles of the converted beams.

The concept of our approach is illustrated in Fig. 1, and described in the section Methods. The laser beam is provided by a linearly polarized Ti:Sa laser system producing ≈35 fs laser pulses with a Gaussian spatial intensity profile. As mentioned above, the main element of our beam-shaping method is the q-plate, a device based on liquid crystal technology^23^,^27^ that is commonly used for generating light beams carrying orbital angular momentum (OAM)^28^. The q-plate essentially works as a birefringent wave plate characterized by an inhomogeneous distribution pattern of the local optic axis in the transverse plane, which is defined by a semi-integer topological charge *q*. Here we exploit a q-plate with *q* = +1/2. Besides the topological charge, the action of the q-plate is determined by the value of the birefringent optical retardation *δ*, that can be controlled electrically by applying an external voltage to the plate^24^. In particular, when acting on a horizontal (vertical) linearly polarized Gaussian beam, the output state is given by:


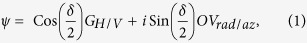


where *G*_*H*/*V*_ stands for the input Gaussian beam with uniform Horizontal or Vertical SoP, respectively, and *OV*_*rad*/*az*_ is an optical vortex (OV) beam with radial or azimuthal SoP. As shown in Eq. (1), the q-plate acts as a transparent medium in the un-tuned condition *δ* = 2π, leaving unchanged the initial Gaussian beam. At the optimal tuning, *δ* = π, it yields standard optical vortex (OVs) beams carrying an OAM 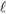 = ±1^28^. After the q-plate, these OV beams present a spatial intensity distribution characterized by a central region of zero intensity (due to undefined phase on the beam axis), a principal intense annulus and several secondary rings at increasing radial distance from the axis^27^,^29^. The central part of these beams is spatially filtered with an iris, thus obtaining OV beams with an annular spatial profile. The SoP of these OV beams can be varied by appropriate tuning of the polarization of the input Gaussian beam^11^,^28^. As an example, Fig. 1(b) reports a schematic of the configuration generating OV beams with radial SoP. Examples of these two cases are schematically illustrated in the upper and lower panel of Fig. 1(b), respectively, with the corresponding spatial profiles of the intensity recorded after the q-plate. In all these cases, the Gaussian and OV beams present a cylindrically symmetric distribution of the SoP and intensity with respect to the optical axis. This, in turn, is reproduced in the surface patterns generated by direct fs laser structuring on the target with OV beams, as already investigated in previous works^11^,^12^,^16^.

As shown in Eq. (1), the q-plate however offers another degree of freedom. When driven by a voltage different from those leading to the tuned (*δ* = π), and un-tuned (*δ* = 2π) conditions illustrated above, the beam generated by the q-plate is a coherent superposition of two fundamental optical states, the Gaussian and OV beams, with relative contributions that vary as the optical retardation changes^22^. An example is schematically shown in the central panel of Fig. 1(b). This, in turn, leads to a class of fs laser beams characterized by an inhomogeneous and asymmetric distribution of polarization and fluence, of which Gaussian and OV beams are the two limiting cases. Here we explore direct fs laser surface structuring induced by focusing these generalized vector beams with a low numerical aperture (NA) lens on a silicon target. Surface structures formed on silicon plates are compared with the simulated polarization and intensity distributions at the lens focal plane of the field reported in Eq. (1). This indeed can be computed at any propagation distance if considering the complete expression of OV beams generated by a q-plate. We omit further details here as a complete theoretical and experimental analysis of these kind of beams is provided in ref. 22.

The surface structuring experiments are carried out by focusing the generated vector beams on the silicon plate, in air. The pulse energy, *E*_0_, is varied by means of a system of half-wave plate and polarizing beam splitter, while the number of pulses hitting the target surface, *N*, is selected by an electromechanical shutter (see Fig. 1(a)). The surface patterns are, then, characterized by a field-emission scanning electron microscope (SEM). SEM images of the sample surface are registered by recording secondary electrons (SE) with an Everhart-Thornley detector. In some cases, SEM images are also registered by using an In-Lens (IL) detector located inside the electron column of the microscope and arranged rotationally symmetric around its axis that collects the SE with high efficiency. The IL detector provides SEM images with higher contrast that are used to obtain zoomed views of sample portions by imaging at higher magnification.

### Surface patterns produced with generalized vector beams generated by the q-plate

**Figure 2:**
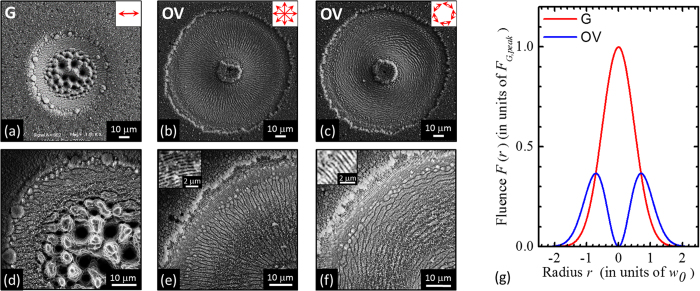
Panels (a–c) are examples of SEM images acquired with the SE detector showing the surface morphologies developed on the silicon target after an irradiation sequence of *N* = 200 pulses at a pulse energy *E*_*0*_ = 45 μJ for the Gaussian beam (left panel, un-tuned q-plate at *δ* = 2π) and the OV beams (central and right panels, tuned q-plate at *δ* = π, radial and azimuthal SoP, respectively). The panels (d–f) are SEM images acquired at higher magnification with the IL detector illustrating the finer details of the surface texture for the three cases. The two insets in panel (e) and (f) are zoomed views of the ripples generated in the peripheral, annular regions at lower fluence of the OV beams. Panel (g) shows the spatial profile of the laser fluence *F*(*r*) as a function of the radius *r* along the diameter of the beam (in units of the beam waist *w*_0_) for a Gaussian (G) and an OV beam with the same pulse energy. The profiles are normalized to the peak fluence of the Gaussian beam *F*_*G,peak*_.

Before analysing fs direct laser structuring of silicon with the vector beams generated by the q-plate at various values of *δ*, it is worth illustrating the surface patterns generated in the two limiting cases of un-tuned and tuned q-plate corresponding, respectively, to the Gaussian (G) and OV components of the generalized vector beams for a fixed value of the total pulse energy. In a recent report, we compared the same two cases for a fixed value of the peak fluence, and found that surface structures locally produced at the same fluence level and number of pulses are characterized by rather similar morphological features^30^. However, the present analysis is carried out for a fixed energy, as opposed to a fixed peak-fluence level, so we need to address these two cases again. Panels (a–d) of Fig. 2 report examples of SEM images of the target surface after an irradiation sequence of *N* = 200 laser pulses at a pulse energy *E*_*0*_ = 45 μJ. The upper panels (a–c) illustrates the different size and morphology of the craters formed on the silicon target, while the lower ones (d–f) show zoomed views registered with the IL detector evidencing more details of the surface structures. Moreover, panel (g) reports the spatial distribution of the laser pulse fluence as a function of the radial coordinate, *r,* which is well described by the expressions^17^,^18^:


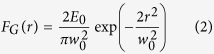



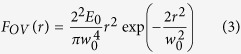


for the G and the OV beams with OAM 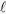 = ±1, respectively, where *w*_0_ is the waist of the fundamental Gaussian beam. At the same energy *E*_0_, the laser fluence of the two beams shows rather different spatial profiles and also different values of the peak fluence. In the present case, the pulse energy is *E*_0_ = 45 μJ. Therefore, the Gaussian beam has a peak fluence 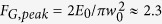 J/cm^2^ at the beam centre (*r* = 0). Instead, the OV beam has a null fluence at the centre (*r* = 0), and the peak fluence occurs at the radial position 
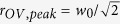
. The corresponding maximum value of the OV beam fluence is *F*_*OV*,*peak*_ = *e*^−1^ × *F*_*G*,*peak*_ ≈ 0.37*F*_*G*,*peak*_, hence *F*_*OV,peak*_ ≈ 0.85 J/cm^2^. This leads to the rather different characteristics observed for G and OV beams in the SEM images of Fig. 2, since the size of the ablation crater and the morphology of the surface structures critically depend on the local value of the laser fluence^3^,^4^,^5^,^6^.

The G beam produces a smaller crater with an external radius of ≈37 μm, while OV beams generate larger annular craters characterized by internal and external radii of ≈10 μm and ≈60 μm, respectively. Moreover, Fig. 2(a) and (d) show that, at the high peak fluence achieved with the Gaussian beam, the crater is characterized by two principal regions presenting different surface textures. The external region for *r* > 25 μm, mainly shows the formation of grooves, while the central region at higher fluence is characterized by coarser micro-wrinkles decorated with several columnar structures and deep cavities, whose typical size is in the range 3–5 μm. This kind of surface structures typically forms in high laser fluence regions or after large number of pulses. In fact, craters produced by reducing the laser peak fluence shows a progressive reduction of the central area characterized by coarser wrinkles followed by an annular grooved region eventually surrounded by an external rippled area.

The OV beam shows the formation of a shallower annular crater reflecting the beam shape and characterized by various regions. In the central region of the beam at null or very low fluence (*r* < 10 μm) an almost unprocessed area decorated with nanoparticles is formed. The ablated crater shows an inner region with a width of ≈30 μm characterized by grooves aligned along the beam polarization. Besides, in the higher fluence region around the OV beam peak (20 μm < *r* < 30 μm), these grooves are partially smashed, suggesting that irradiation at larger fluence with a high number of shots can be responsible of a progressive modification of the grooves morphology as well as of the columnar structures observed in the case of the G beam. According to the spatial profiles of the fluence, columnar structures mainly form at fluence values larger than ≈0.9 J/cm^2^ (for *N* = 200), therefore they are not recognizable in the craters generated by the OV beam due to its lower peak fluence. At the lower fluence values attained in the external periphery of the OV beam (e.g. for 10 μm < *r* < 13 μm and 48 μm < *r* < 60 μm), subwavelength ripples are formed in two annular regions surrounding the grooved area.

The preliminary characterization reported above suggests that the various regions of the beam spot on the silicon target can be qualitatively graded according to their surface morphology, thus associating the coarse structures with columnar morphology, the grooves and the ripples to a large, intermediate and low fluence range, respectively. Moreover, the preferential orientation of the surface structures provides direct clues on the local SoP of the beam.

We turn now to the case of the generalized vector beams obtained for intermediate tuning and to the main characteristics of the crater shape and surface patterns they produce on the silicon target. Several fs vector beams are generated by varying the optical retardation of the q-plate, *δ*. The theoretical spatial profiles of the laser fluence and SoP in the focal plane are obtained by simulating the optical field propagation, as described in ref. 22.

**Figure 3:**
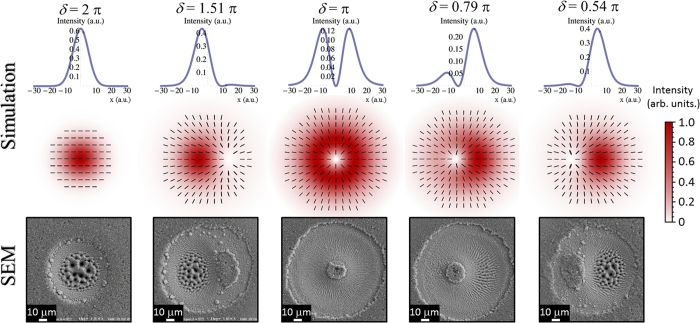
The central panels show the spatial profiles, in the focal plane, of both the fluence and the SoP of various vector beams generated by changing the value of the q-plate optical retardation *δ*. The experimental configuration corresponds to that leading to a radially polarized OV beam at optimal tuning of the q-plate optical retardation *δ* = π. For each panels, the fluence is normalized to its own maximum value according the false color scale reported on the right. Moreover, the polarization ellipses defining the SoP in each location of the generalized vector beams are very narrow, i.e. the local polarization is approximately linear and well approximated by a segment indicating the orientation of its dominant component. The upper panels report the corresponding one-dimensional fluence profiles along the horizontal diameter. The lower panels show SEM images of the corresponding craters produced on the silicon target surface after an irradiation sequence of *N* = 200 pulses at a pulse energy *E*_0_ = 45 μJ, registered with the SE detector.

Let us consider first the case of a generalized vector beam after the q-plate consisting in a superposition of a Gaussian beam with horizontal polarization and an OV beam with radial SoP, as illustrated in Fig. 1(b). Figure 3 illustrates the variation of the beam characteristics by reporting examples of intensity and SoP spatial profiles at various values of *δ*. In particular, for each value of *δ*, the central panel reports a two-dimensional map that shows the SoP (ellipses) and the fluence (intensity, in false colour) spatial distributions of the laser beam. It is worth noting that the polarization ellipses defining the SoP in each location of the beam are very narrow, that is the SoP is approximately linear, and well approximated by a segment whose orientation indicates the dominant local component of the polarization. Moreover, the upper panel shows the one-dimensional profile of the fluence spatial distribution along the horizontal axis passing through the beam centre. As anticipated, in the focal plane the fs vector beam is described as a superposition of two fundamental components: the OV and G beams corresponding to the tuned and un-tuned q-plate discussed above. This property is clearly addressed by the maps reported in the central panels of Fig. 3. In particular, the optical retardation tuning produces a change of the relative contributions and a spatial separation of the two components with a shift of the position of the region of minimum fluence of the beam along the q-plate axis, which in Fig. 3 is horizontal. Finally, the lower panels of Fig. 3 show SEM images of the silicon target surface after an irradiation sequence of *N* = 200 pulses at a laser energy *E*_0_ = 45 μJ.

**Figure 4:**
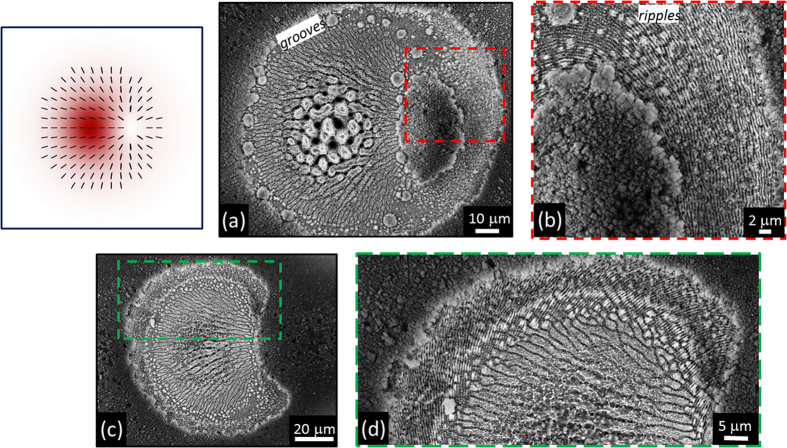
(**a**) SEM image, acquired with the IL detector, illustrating the surface morphology developed on the silicon target after an irradiation sequence of *N* = 200 pulses at a pulse energy *E*_0_ = 45 μJ, for the radial vector beam generated at *δ* = 1.51 π. (**b**) Zoomed view of the area indicated by the red dashed box in panel (a). (**c**) SEM image, acquired with the IL detector, illustrating the surface morphology developed on the silicon target after an irradiation sequence of *N* = 200 pulses at a pulse energy *E*_*0*_ = 27 μJ at *δ* = 1.51 π. (**d**) Zoomed view of the area indicated by the green dashed box in panel (c). Upper-left inset: map of the SoP and fluence of the radial fs vector beams generated at *δ* = 1.51 π.

Besides a perfectly G beam with uniform horizontal polarization at *δ* = 2 π and the radially polarized OV beam at *δ* = π, a variety of fs vector beams with a prevalent radial SoP can be generated, which we indicate as radial vector beams. The examples in Fig. 3 show two cases of radial fs vector beams with the region of minimum fluence located off-axis at the two opposite sides with respect to the beam centre for *δ* = 1.51 π and *δ* = 0.54 π, respectively. Moreover, radial vector beams characterized by a small shift of the position of the region of minimum fluence and a slight asymmetric distribution of the fluence and SoP are also achieved, as e.g. at *δ* = 0.79 π. The SEM images of Fig. 3 demonstrate that the shapes of the ablation craters closely reflect the variation of the fluence spatial distribution of the various fs vector beams. Voltage tuning also influences the SoP of the fs vector beam allowing to create optical states with an asymmetric spatial distribution of the polarization direction for values of *δ* different from π and 2 π. The corresponding effect on the surface structures is illustrated in Fig. 4(a) and (b), which report SEM images of the crater produced at *δ* = 1.51 π and registered with the IL detector. In particular, Fig. 4(b) is a zoomed view of a part of the crater, close to the region of minimum fluence, that better evidences the spatial arrangement of the surface ripples. In Fig. 4(a) one can easily appreciate the fairly good correspondence between the surface structures and the fs vector beam map, reported in the upper-left inset for easiness of comparison. In particular, the SEM image shows an area characterized by columnar structures and coarse micro-wrinkles located in the higher fluence region in the centre-left part of the beam. This area is surrounded by a pattern of grooves displaying a preferential arrangement along the local direction of the beam polarization in the area of the beam characterised by intermediate values of the fluence. Opposite to the high intensity area, an elliptically shaped, nearly unprocessed area decorated with nanoparticles is formed in the part corresponding to the lower intensity region of the beam^13^. This area is slightly elongated in the vertical direction resembling the asymmetric shape of the region of minimum fluence present in the corresponding radial vector beam map (Fig. 4, upper-left inset). Around this region, a rippled zone corresponding to rather low fluence values is recognized in the zoomed view of Fig. 4(b). These ripples are preferentially ordered along the direction of the normal to the local polarization of the radial vector beam.

Figure 4(c) addresses the variation of the crater shape when the energy of the radial fs vector beam (*δ* = 1.51 π) is reduced to *E*_*0*_ = 27 μJ. Due to the lopsided distribution of the laser intensity, bow-shaped craters, as the one exemplified in Fig. 4(c) and that resembles the figure of a half-moon, are generated on the target surface. The zoomed view of a portion of this crater is reported in Fig. 4(d) to evidence the arrangement of the various surface structures: ripples, well-developed grooves and smashed grooves progressively appear going from the crater edges, at lower fluence, towards the more intense part of the beam. Craters with a shape nearly specular with respect to the vertical to the one of Fig. 4(c) are obtained when tuning the voltage to a value close to *δ* = 0.54 π (not shown) as a consequence of both the redistribution of the laser intensity and the shift of the position of the region of minimum fluence (see Fig. 3).

**Figure 5:**
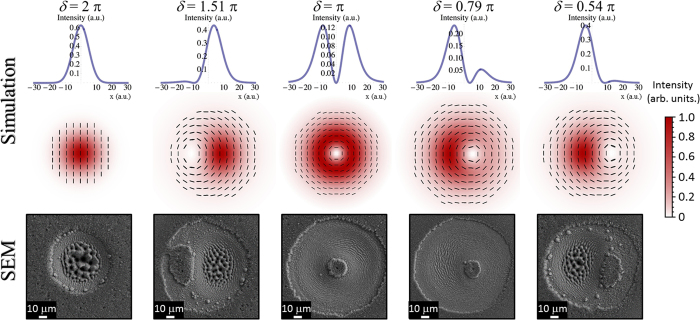
Same as for Fig. 3 for an experimental configuration corresponding to that leading to an azimuthally polarized OV beam at optimal tuning of the q-plate optical retardation *δ* = π.

We turn now to the case of an OV beam with an azimuthal SoP at optimal tuning of the q-plate (*δ* = π). Likewise the previous case, we name the generated singular beams as azimuthal fs vector beams. Figure 5 reports examples of the beam properties generated by varying the value of *δ*. At *δ* = 2 π, a perfect G beam with uniform vertical polarization is produced. Then, several asymmetric, azimuthal vector beams like the one shown in Fig. 5 for *δ* = 1.51 π are progressively generated reducing the values of *δ*, finally approaching the perfect azimuthally polarized OV beam at *δ* = π. As *δ* is further decreased, other azimuthal fs vector beams are obtained with a continuous rightward shift of the region of minimum fluence. Interestingly, the azimuthal fs vector beams show a displacement of the region of minimum fluence in a reverse direction with respect to radial fs vector beams as a function of the q-plate optical retardation (see Figs 3 and 5). Moreover, the fluence spatial profiles of the azimuthal and radial fs vector beams, at the same value of *δ*, are specular with respect to the vertical line passing through the location of the G or OV beams centres.

**Figure 6:**
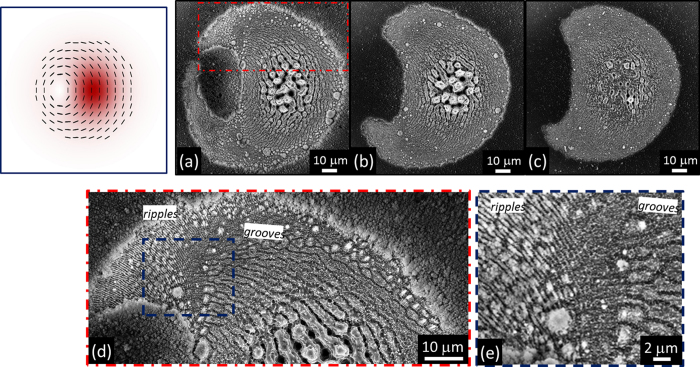
SEM images, acquired with the IL detector, illustrating the surface morphology developed on the silicon target after irradiation sequences of (**a**) *N* = 200, (**b**) *N* = 100 and (**c**) *N* = 50 pulses, respectively, at a pulse energy *E*_0_ = 45 μJ, for the azimuthal fs vector beam generated at *δ*  = 1.51 π. (**d**) Zoomed view of the area indicated by the red dashed box in panel (a). (**e**) Zoomed view of the area indicated by the blue dashed box in panel (d). Upper-left inset: map of the SoP and fluence of the azimuthal fs vector beam generated at *δ* = 1.51 π.

Also for azimuthal vector beams, the SEM images of the target surface reported in the lower panels of Fig. 5 show SEM images of the silicon target surface after an irradiation sequence of *N* = 200 pulses at a laser energy *E*_0_ = 45 μJ. Panels (a)–(c) of Fig. 6 address the change of the crater shape as a function of the number of pulse *N* for an azimuthal fs vector beam (*δ* = 1.51 π) at an energy *E*_0_ = 45 μJ. The progressive reduction of *N* leads to the gradual formation of half-moon shaped craters as a consequence of the asymmetric distribution of the laser fluence. Moreover, the various surface structures become vaguer and less defined for lower number of pulses. Figure 6(d) and (e) report SEM images acquired at higher magnification of portions of the crater shown in Fig. 6(a) addressing the arrangement of the various surface structures produced by azimuthal fs vector beams. The surface structures display a good correlation with the fs vector beam map shown in the upper-left inset of Fig. 6 to facilitate the comparison. The region corresponding to the more intense part of the beam presents the characteristic coarser wrinkles decorated by columnar structures. This region is enclosed by an array of well-defined grooves displaying a preferential azimuthal orientation in the areas of the fs vector beam at intermediate values of the fluence, eventually verging towards the elliptically shaped, nearly unprocessed area decorated with nanoparticles located in the sector corresponding to the region of minimum fluence of the laser beam. Finally, ripples preferentially ordered along the direction of the normal to the local polarization of the azimuthal fs vector beam cover the low intensity part of the beam, as shown in Fig. 6(e).

## Discussion

The use of ultrashort laser beams with inhomogeneous spatial distribution of the polarization reveals to be an extraordinary way to handle very complex and rich structural surface patterns with relatively few control parameters (*N, E*_0_, *δ*). This control can be achieved remotely and programmatically, thus opening the route to fabricate more complex surface structures by direct fs laser surface structuring. Here, an experimental approach based on a q-plate was used to demonstrate the generation of a variety of novel ultrashort generalized vector beams, by exploiting for the first time the further degree of freedom offered by the q-plate driving voltage in the context of laser surface structuring. Two experimental configurations were used in our investigation leading to the generation of radial and azimuthal fs generalized vector beams (see Figs 1, 3 and 5). In particular, our beam generator allows getting Gaussian beams with linear polarization for an un-tuned q-plate, while radial or azimuthal OV beams are produced at the optimal tuning conditions. These fundamental kinds of optical beams can be used to form rather regular surface patterns on the silicon target surface, as illustrated in Fig. 2. The surface structures show a multiplicity of forms and arrangements like sub-wavelength ripples, supra-wavelength grooves and coarse micro-wrinkles decorated by columnar structures according to the local level of excitation and SoP.

By tuning the q-plate optical retardation, a class of generalized vector beams is generated through a coherent superposition of the two fundamental beam states in the focal plane. Examples of the various fs vector beams that can be achieved are reported in the central panels of Figs 3 and 5, which show maps of the SoP and fluence spatial distributions of the beam. The SEM images of Figs 3 and 5 report several examples of the crater shapes and surface structures that can be fabricated by direct laser surface structuring with the fs vector beams. For instance, the variation of the optical retardation through voltage tuning allows one to shift the position and vary the size of the region of minimum fluence of the vector beams, to bend the direction of the polarization and to spatially redistribute the beam energy. These effects directly influence the characteristics of the produced craters and the resulting surface structures morphologies. Particularly interesting is the possibility of generating lopsided craters also in the form of a half-moon by appropriate selection of the laser pulse energy and/or number of pulses, as shown in Figs 4 and 6, as well as controlling its orientation by means of the polarization of the fs vector beams. These features further extend the kind of surface structures that can be achieved by direct fs laser irradiation also establishing the q-plate beam converter as a facile and versatile way to generate optical beams with ultrashort pulse duration characterized by an asymmetric spatial distribution of the SoP and fluence for laser processing applications.

**Figure 7:**
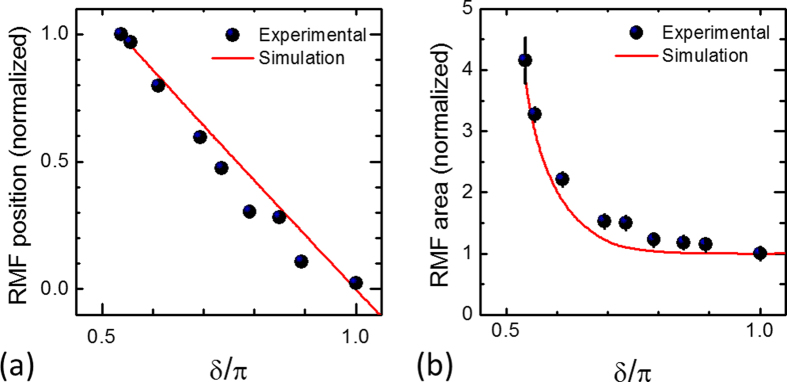
Variation of the central position (**a**) and area (**b**) of the region of minimum fluence as a function of the optical retardation *δ*. The experimental values of the region of minimum fluence correspond to the central position and area of the nearly unprocessed area decorated with nanoparticles present in the crater produced on the target surface. The corresponding simulation values represent the location of the minimum beam fluence and the area of a region of the beam delimited by fixing an appropriate fraction of the peak fluence coherent with the experimental value of the ablation fluence threshold. In panel (a), the error bars are contained within the data point symbols.

In all cases, we observe a rather good correspondence between the state of the optical beam and the experimentally observed surface structure. In an attempt to underline such reliable consistency, in Fig. 7 we report as solid dots experimental data obtained by estimating the central position (panel (a)) and the area (panel (b)) of the nearly unprocessed region decorated with nanoparticles that identifies the region of minimum fluence in the crater produced on the target surface. The corresponding values for the beam are reported as solid lines in Fig. 7. Since the removal of material in the ablation process is a threshold phenomenon, the area of the region of minimum fluence of the beam is derived from the map of the fluence spatial profile by fixing an appropriate fraction of the peak fluence coherent with the experimental case. Moreover, the variation of the central position of the region of minimum fluence corresponds to the shift of the location of the minimum of the beam fluence spatial profile. The data in Fig. 7(a) are normalized to the maximum value achieved by the shift of the region of minimum fluence position corresponding to *δ* = 0.54 π, while in Fig. 7(b) the area of the region of minimum fluence is normalized to the minimum value achieved at optimal tuning (*δ* = π). Due to the symmetric behavior with respect to the optimal tuning condition, the data are only reported for *δ* ≤ π. Moreover, data for values lower than *δ* = 0.54 π are not considered because the region of minimum fluence shifts in areas of very low fluence which makes it not possible identifying the corresponding area in the crater generated on the target surface (see e.g. Fig. 4 (c)). Figure 7 shows that the simulation results reproduce fairly well the observed experimental trend, thus indicating that the predicted features of the fs vector beams are very consistent with the main characteristics of the craters formed on the silicon target. In addition, the accurate matching between the directional arrangements and spatial distribution of the produced surface structures and the SoP and fluence distribution of the fs vector beams suggest that analysis of ablation craters and surface structures can be effectively used as a profiling method to diagnose intense, complex ultrashort laser beams.

In conclusion, we have demonstrated that a beam converter based on a q-plate can be effectively exploited to generate fs generalized vector beams and experimentally investigated the application of these optical beams to direct surface structuring of silicon. Our findings single out the possibility of using the voltage tuning of the q-plate beam converter to vary its optical retardation *δ*, thus generating several complex surface structures decorated with patterns of ripples, grooves or more complex forms directly associated to the local state of the optical vector beam. Moreover, the direct association between the various features of the observed surface structures and the local state of the fs vector beam suggests that direct analysis of ablation craters can be a valuable way to diagnose complex ultrashort laser beams. Our results evidence that an appropriate tuning of the level of excitation achieved through a suitable selection of the energy and number of laser pulses can lead to the elaboration of asymmetric shaped craters and lopsided distributions of the surface structure. While our investigation was limited to fs vector beams generated with a q-plate with a topological charge *q* = +1/2 in two specific configurations (i.e. radial and azimuthal OV beams at optimal q-plate tuning), other experimental arrangements of the q-plate beam converter, as for example higher values of *q* or OV beams with even more complex SoP at optimal tuning, can be designed and used to fabricate still more complex surface micro-structures. Since the formation of surface structures seems to be ubiquitous to laser irradiation of solid targets with ultrashort pulses, the method we describe can be directly extended to other materials of interest. Finally, the possibility of fast switching intrinsic to a voltage tuning of the state of the q-plate can be joined to switchable wave-plates, as e.g. electronic controlled liquid crystal retarders, allowing the implementation of a setup based on high-speed generation and tuning of fs vector beams for an efficient fabrication of complex arrays of surface structures based on direct fs laser processing of solid targets. All these aspects will be subject of future research work.

## Methods

### Experimental setup

The experimental setup is sketched in Fig. 1(a). The laser source is a Ti:Sa laser system delivering ≈35 fs pulses at a central wavelength of 800 nm with a Gaussian beam spatial profile, at a repetition rate of 100 Hz. Optical vector beams are generated by a beam converter based on a q-plate with a topological charge *q* = +1/2. The q-plate tuning is achieved by varying the optical retardation *δ* by means of the driving voltage V_pp_ (peak to peak) applied to the q-plate by using a square-wave at 11 kHz delivered by a signal generator. At the optimal tuning, corresponding to a half-wave retardation (*δ* = π), the q-plate allows generating optical vortex (OV) beams carrying an orbital angular momentum (OAM) 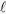 = ±1 through spin-to-orbital conversion of the angular momentum of light. An example of OV beam with a radial state of polarization (SoP) is shown in the lower panel of Fig. 1(b). A full wave retardation (*δ* = 2 π) results in a Gaussian beam at the q-plate output, as illustrated in the upper panel of Fig. 1(b)). The fluence profile of the un-tuned case (*δ* = 2 π) corresponding to a Gaussian beam is described by Eq. (2) and illustrated in Fig. 2(g). The OV beams generated in the tuned case (*δ* = π) are characterised by an annular spatial profile with a central region of zero intensity due to an undefined phase on the OV beam axis, as described by Eq. (3) and illustrated in Fig. 2(g). In our experimental conditions, the beam waist is *w*_0_ ≈ 35 μm.

Partial tuning of the q-plate allows generating light beams, here indicated as fs generalized vector beams, for which only a partial conversion to an OV state is achieved. Therefore, these beams can be described as a superposition of an OV and a Gaussian beam (see Eq. (1)), with a variable fraction of these two components. When focused with a low NA lens, as in our experimental conditions, these two components become spatially shifted in the focal plane. This, in turn, allows generating lopsided, fs laser beams with a rather complex spatial variation of the SoP and fluence distribution. An example of such a vector beam is schematically reported in the central panel of Fig. 1(b).

The target is an intrinsic (resistivity >200 cm), single-crystalline Si (100) plate (dielectric constant ε_Si_ = 13.64 + 0.048*i* at 800 nm). The laser beam is focused by a lens (focal length 75 mm) onto the Si target sample mounted on a computer-controlled XY-translation stage at normal incidence. An electromechanical shutter controls the number of laser pulses, *N*, irradiated on the target surface. The morphological modification of the irradiated target surface is analysed by means of a field emission scanning electron microscope (FESEM).

## Additional Information

**How to cite this article**: JJ Nivas, J. *et al*. Surface Structuring with Polarization-Singular Femtosecond Laser Beams Generated by a q-plate. *Sci. Rep.* **7**, 42142; doi: 10.1038/srep42142 (2017).

**Publisher's note:** Springer Nature remains neutral with regard to jurisdictional claims in published maps and institutional affiliations.
